# Anti-inflammatory effects of nicotinic acid in adipocytes demonstrated by suppression of fractalkine, RANTES, and MCP-1 and upregulation of adiponectin

**DOI:** 10.1016/j.atherosclerosis.2009.08.045

**Published:** 2010-03

**Authors:** Janet E. Digby, Eileen McNeill, Oliver J. Dyar, Vincent Lam, David R. Greaves, Robin P. Choudhury

**Affiliations:** Department of Cardiovascular Medicine (J.E.D., E.M., O.J.D., V.L., R.P.C.) and Sir William Dunn School of Pathology (D.R.G.), Oxford, University of Oxford, United Kingdom

**Keywords:** Nicotinic acid, Niacin, Adipocyte, Chemokine, Inflammation, Adipose tissue

## Abstract

**Objective:**

A major site of action for the atheroprotective drug nicotinic acid (NA) is adipose tissue, via the G-protein-coupled receptor, GPR109A. Since, adipose tissue is an active secretory organ that contributes both positively and negatively to systemic inflammatory processes associated with cardiovascular disease, we hypothesized that NA would act directly upon adipocytes to alter the expression of pro-inflammatory chemokines, and the anti-inflammatory adipokine adiponectin.

**Methods and results:**

TNF-α treatment (1.0 ng/mL) of 3T3-L1 adipocytes resulted in an increase in gene expression of fractalkine (9 ± 3.3-fold, *P* < 0.01); monocyte chemoattractant protein-1 (MCP-1) (24 ± 1.2-fold, *P* < 0.001), ‘regulated upon activation, normal T cell expressed and secreted’ (RANTES) (500 ± 55-fold, *P* < 0.001) and inducible nitric oxide synthase (iNOS) (200 ± 70-fold, *P* < 0.05). The addition of NA (10^−4^ M) to TNF-α-treated adipocytes attenuated expression of fractalkine (50 ± 12%, *P* < 0.01); MCP-1 (50 ± 6%, *P* < 0.01), RANTES (70 ± 3%, *P* < 0.01) and iNOS (60 ± 16%). This pattern was mirrored in protein released from the adipocytes into the surrounding media. The effect on gene expression was neutralised by pre-treatment with pertussis toxin. NA attenuated macrophage chemotaxis (by 27 ± 3.5%, *P* < 0.001) towards adipocyte conditioned media. By contrast, NA, (10^−6^–10^−3^ M) increased, in a dose-dependent manner, mRNA of the atheroprotective hormone adiponectin (3–5-fold *n* = 6, *P* < 0.01).

**Conclusions:**

NA suppresses pro-atherogenic chemokines and upregulates the atheroprotective adiponectin through a G-protein-coupled pathway. Since adipose tissue has the potential to contribute to both systemic and local (perivascular) inflammation associated with atherosclerosis our results suggest a new “pleiotropic” role for NA.

## 1 Introduction

There is ample epidemiological evidence linking obesity, and the related ‘metabolic syndrome’, with vascular disease [1]. Conventionally, adipose tissue has been regarded as an inert store of triglycerides and fatty acids, but there is accumulating evidence that adipose tissue is involved in more diverse activity, including pro-inflammatory processes [2]. Several secreted factors, termed ‘adipokines’ influence local and distant inflammatory processes. For instance, mature adipocytes upregulate the transcriptional regulator NF-κB leading to secretion of interleukin-6 (IL-6) and tumour necrosis factor-α (TNF-α) [3] and to recruitment of macrophages [4]. Mesenteric adipose tissue has been associated with distant atherosclerosis, assessed by measurement of carotid intima thickness using ultrasound [5]. Similarly abdominal visceral fat appears to be associated with aortic [6] and carotid artery [7] stiffness and with elevated IL-6 and CRP, which have been postulated as mediators [7].

Furthermore, there is emerging evidence that perivascular adipose tissue influences vascular function and may have the potential to alter susceptibility to atherosclerosis in adjacent arteries in a paracrine manner [8]. Mazurek et al. found greater expression of pro-inflammatory cytokines in epicardial adipose tissue than in subcutaneous fat in patients undergoing coronary artery bypass grafting [9], while Henrichot et al. have identified pro-inflammatory cytokines IL-8 and monocyte chemoattractant protein-1 (MCP-1) in human peri-aortic white adipose tissue and demonstrated the potential of this tissue to promote recruitment of peripheral blood leucocytes [10].

Adipose tissue is an important target for nicotinic acid [11]. A G-protein-coupled receptor (GPCR), that binds NA, GPR109A has recently been given the HGNC approved gene symbol, NIACR1 but is also termed HM74a, in humans and ‘protein upregulated in macrophages by interferon-gamma’ or ‘PUMA-G’, in mice [12,13]. Activation of the receptor in adipocytes inhibits lipolysis via G_i_ mediated effects on adenylate cyclase, with decreased cellular cAMP levels [12], reduced lipolysis and a reduction in free fatty acids flux to the liver as substrate for VLDL synthesis. In patients nicotinic acid reduces LDL-cholesterol and increases HDL-cholesterol [14]. The observed reduction in the progression of atherosclerosis and cardiovascular morbidity with NA may be due solely to its lipid modifying effects [15]. However, niacin treatment has also been shown to increase plasma levels of the adipocyte-derived atheroprotective hormone, adiponectin [16] raising the possibility that some of the effects of NA may be mediated through lipid-independent pathways. Here, we show that NA acts directly upon adipocytes to reduce the expression of pro-inflammatory chemokines; fractalkine, MCP-1 and RANTES with inhibitory effects on monocyte chemotaxis, but with increase in the atheroprotective adipokine, adiponectin.

## 2 Materials and methods

### 2.1 Cell culture

3T3-L1 preadipocytes (ATCC, Teddington, UK) were seeded in 6-well plates at a density of 10^5^ per well and cultured with Dulbecco's modified Eagle's medium (DMEM) supplemented with, L-glutamine (4 mM), 10% fetal calf serum, penicillin, 100 IU and streptomycin, 100 μg/mL, in a humidified atmosphere of 95% air/5% CO_2_ at 37 °C. At confluence, cells were differentiated into adipocytes by the addition insulin (100 nM) and dexamethasone (100 nM). Morphological analysis showed that typically 80 to 90% of cells had differentiated by 10 days of incubation with differentiation media. All cell culture reagents were purchased from Sigma Aldrich (Poole, UK).

### 2.2 Cell treatments

Prior to treatments, cells were cultured in media without insulin and dexamethasone for 24 h then serum starved for 4 h. Cells were treated for 4 or 24 h with nicotinic acid (10^−3^–10^−^6 M, Sigma Aldrich, Poole, UK), and/or TNF-α 0.1, 0.5 1,0 or 10 ng/mL (R and D Systems, Abingdon, UK). For the pertussis treatments, cells were incubated with pertussis toxin (Calbiochem, MERK, Nottingham, UK) for 16 h to activate the toxin, then treated with nicotinic acid and/or TNF-α for 4 h. At the end of the incubation times, the surrounding cell culture media, ‘adipocyte conditioned media’ (ACM) was collected and snap frozen then stored at −80 °C until analysis. Cells were lysed on ice in RNA lysis buffer supplied with the Qiagen RNEasy mini kit (Qiagen, Crawley, UK).

### 2.3 Measurement of adipokines and chemokine gene expression

Total RNA was prepared using Qiagen RNEasy mini columns and 1 μg was reverse transcribed using a QuantiTect^®^ Reverse Transcription Kit using Oligo dT's and random hexamers as primers. Real-time PCR was carried out with 1 μL of cDNA in a 10 μL reaction mix consisting of Sybr Green Mastermix (Applied Biosystems, Warrington, UK) and sense and antisense primers (0.25 μM final concentration). Primer sequences are shown in Table 1. Cycling parameters were as follows: activation of Taq polymerase, 10 min at 95 °C, then 40 cycles at 95 °C for 15 s, then extension at 60 °C for 1 min, followed by a melt curve analysis.

### 2.4 Measurement of secreted adipokines and chemokines

Secreted chemokines, MCP-1, fractalkine, RANTES and adiponectin were measured in the media removed from adipocytes after 24 h incubation by a Luminex™ Multiplex bead-based system using Milliplex™ MAP kits, from the mouse cytokine/chemokine panel according to manufacturer's instructions.

### 2.5 Chemotaxis and chemokinesis assays

To investigate the biological response resulting from of nicotinic acid treatment, we used a chemotaxis assay to measure macrophage migration towards ACM from differentiated treated 3T3-L1 adipocytes. Cells were exposed to TNF-α (1.0 ng/mL) with or without nicotinic acid 10^−3^ M for 24 h, then media collected and stored at −80 °C prior to chemotaxis transwell assays.

Murine macrophages were harvested by peritoneal lavage with PBS and 5 mM EDTA 4–5 days after intraperitoneal injection of 2% Bio-gel in PBS. Chemotaxis was measured using pooled macrophages obtained from two C57/BL6 mice subjected to peritoneal lavage and repeated on three separate experiments.

For the chemotaxis assays, cells were suspended in chemotaxis buffer; RPMI with HEPES (25 mM), and 0.1% BSA, and applied to a 96-well Neuroprobe ChemoTx™ membrane (Receptor Technologies, Adderbury, UK), 8 μM pore size at a density of approximately 400,000 cells per well. The lower chamber contained either 1:3 diluted ACM or chemotaxis buffer alone. As negative controls, wells included chemotaxis migration buffer only, and migration buffer with TNF-α (1.0 ng/mL) to ensure that there was no chemotaxis to TNF-α alone. For the chemokinesis assay, macrophages were suspended in ACM from TNF-α-treated cells and placed on the upper side of the membrane with the lower chamber containing the same concentration of ACM. After 4-h incubation at 37 °C in a 5% CO_2_ cell culture incubator, the cells on the upper layer of the membrane were removed with a cotton swab and the membrane rinsed with PBS. Migrated cells attached to the lower area of the membrane were fixed in paraformaldehyde (4%) then mounted with mounting media containing DAPI. Migration of the cells was quantified by taking 2 images under a fluorescent microscope from each membrane with a minimum of 4 membranes per treatment. Stained nuclei were then counted using image software Image Pro Plus™ (Media Cybernetics, Silver Spring, Maryland).

### 2.6 Cell viability assay

Please see Supplementary methods and figures.

### 2.7 iNOS mRNA expression

Please see Supplementary methods and figures.

### 2.8 Demonstration of GPR109a gene expression in 3T3-L1 adipocytes

Please see Supplementary methods and figures.

## 3 Results

### 3.1 TNF-α increases the expression of chemokines in 3T3-L1 adipocytes

**Fig. 1:**
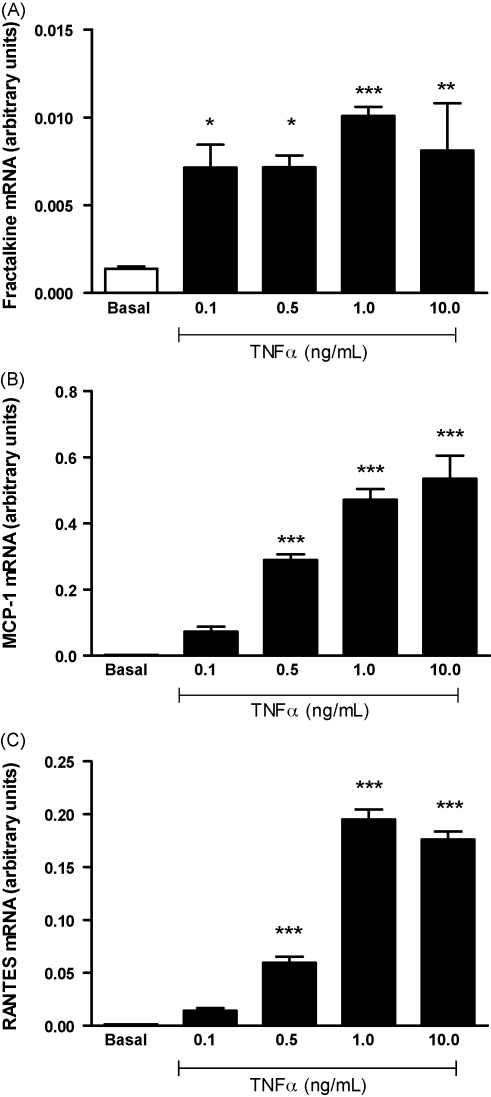
The effect of varying doses of TNF-α on mRNA levels of Fractalkine (A), MCP-1 (B) and RANTES (C) determined by real-time RT-PCR using the 2-ΔΔCT method, normalised to the housekeeping gene cyclophilin. *n* = 6 for each treatment, ^**^P < 0.01, ^***^P < 0.001 via one-way ANOVA with Bonferroni's multiple comparison post-hoc test.

We quantified expressions levels of the CC chemokines, MCP-1 and RANTES and the CX3C chemokine, fractalkine, all of which are chemoattractant factors involved in monocyte recruitment. Expression was measured under basal conditions and after stimulation with varying doses of TNF-α. Non-stimulated 3T3-L1 adipocytes expressed measurable mRNA levels for MCP-1, RANTES and fractalkine. Expression of each was significantly upregulated by exposure to TNF-α. The optimal concentration of TNF-α was established by testing a concentration range of 0.1, 0.5, 1.0 and 10 ng/mL. Maximal chemokine mRNA upregulation in response to TNF-α treatment was achieved using 1.0 ng/mL (Fig. 1, *n* = 6). In addition, a cell viability assay in response to TNF-α and NA treatment was undertaken. TNF-α exposure of ≥10 ng significantly reduced cell viability (see Supplementary on-line Fig. i). Therefore, for subsequent experiments, a concentration of 1.0 ng/mL was used to induce a maximal inflammatory response without causing cell toxicity.

### 3.2 Nicotinic acid suppresses expression and secretion of inflammatory chemokines in 3T3-L1 adipocytes exposed to TNF-α

**Fig. 2:**
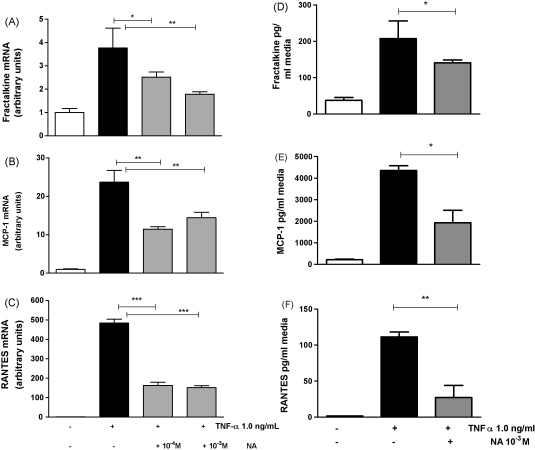
Gene expression and protein secretion. mRNA levels of Fractalkine (A), MCP-1 (B) and RANTES (C) determined by real-time RT-PCR using the 2-ΔΔCT method, normalised to the housekeeping gene cyclophilin. Secreted protein (pg/mL) of fractalkine (D), MCP-1 (E), RANTES (F). For RNA analyses, cells were incubated for 4 h and for media analysis cells were incubated for 24 h, with DMEM only (Basal), DMEM + TNF-α 1.0 ng/mL (TNF-α), DMEM + TNF-α 1.0 ng/mL + nicotinic acid 10^−4^ M (TNF-α + NA^−4^) or DMEM + TNF-α 1.0 ng/mL + nicotinic acid 10^−3^ M (TNF-α + NA^−3^). *n* = 6 for each treatment, ^*^P < 0.05, ^**^P < 0.01, ^***^P < 0.001 via one-way ANOVA with Bonferroni's multiple comparison post-hoc test.

To determine the effect of NA on gene expression and secretion of pro-inflammatory chemokines in differentiated 3T3-L1 adipocytes, cells were exposed to TNF-α (1.0 ng/mL) with or without the addition of NA. At 4 h there were significant increases in mRNA for fractalkine (5 ± 1.3-fold, *P* < 0.01), MCP-1 (24 ± 1.2-fold, *P* < 0.001), and RANTES (500 ± 55-fold, *P* < 0.001) (Fig. 2, *n* = 6). The addition of NA (10^−4^ and 10^−3^ M) to TNF-α-treated adipocytes attenuated expression of fractalkine (40 ± 14%: *P* < 0.01); MCP-1 (50 ± 6%: *P* < 0.01) and RANTES (68 ± 2%: *P* < 0.001).

In media taken from cells incubated for 24 h protein for fractalkine, MCP-1 and RANTES were all significantly increased following treatment with TNF-α 1.0 ng mL (7.6 ± 5.4-fold, 21.8 ± 4.6-fold, *P* < 0.05 and 80.8 ± 4.1-fold, *P* < 0.01, respectively). The addition of NA 10^−3^ M resulted in a reduction in fractalkine, MCP-1 and RANTES protein measured in the media (19.2 ± 5.0%, *P* < 0.05, 54.8 ± 15.4% and *P* < 0.05, 74 ± 15%, *P* < 0.01, respectively). (Fig. 1D–F, *n* = 4.)

As inducible nitric oxide synthase (iNOS) is also strongly affected by exposure to TNF-α in adipose tissue and is involved in activation of inflammatory pathways [17] the effects of niacin on iNOS mRNA expression levels were tested in this cellular model. iNOS mRNA levels measured in untreated cells was almost undetectable, however, treatment with TNF-α (1.0 ng/mL) for 4 h resulted in a significant upregulation of gene expression. The addition of NA (10^−4^ and 10^−3^ M) to TNF-α treated adipocytes again attenuated expression of iNOS mRNA (63 ± 21% and 160 ± 17%). This effect was abolished by pre-treatment with pertussis. Details of methods and results are shown in Supplementary methods and Fig. ii.

### 3.3 Inhibition of G-protein-coupled receptor signalling by pertussis abolishes the inhibitory effect of nicotinic acid on chemokine expression

**Fig. 3:**
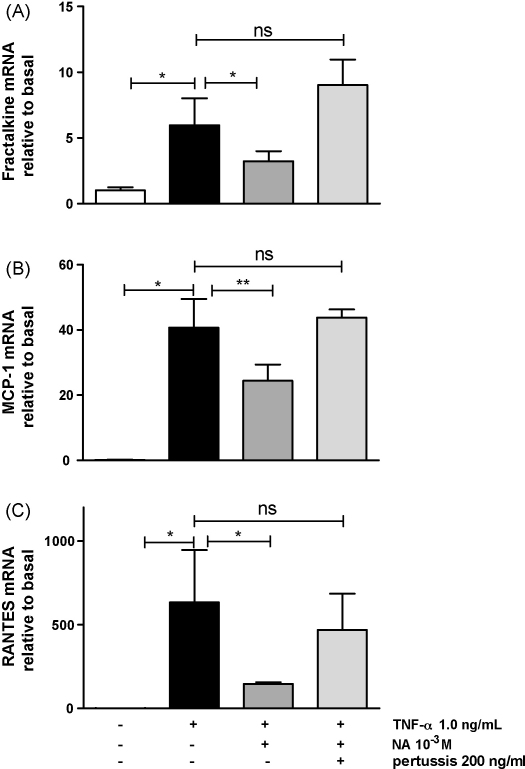
The effect of pre-treatment with pertussis (PTX) on adipocyte mRNA expression of fractalkine (A), MCP-1 (B) and RANTES (C) treated with TNFα and NA. *n* = 6 for each treatment, ^**^P < 0.01, ^***^P < 0.001 via one-way ANOVA with Bonferroni's multiple comparison post-hoc test.

To assess whether the anti-inflammatory effects of NA were mediated by G_i_-protein-coupled receptor (GPCR) signalling, these experiments were repeated following pre-incubation with pertussis toxin (PTX.) which uncouples GPCR signalling form G_i_ and G_o_. Pre-incubation with PTX (100 ng/mL) for 18 h prior to TNF-α and NA treatment abolished the observed reduction in gene expression of MCP-1, RANTES and fractalkine (Fig. 3, *n* = 6). Similar effects were demonstrated with iNOS expression, see Supplementary Fig. ii(B).

### 3.4 GPR109a gene expression and the effect of exposure to TNF-α

Gene expression of the G-protein-coupled receptor, GPR 109a was measured using quantitative RT-PCR and was 100-fold greater in 3T3-L1 adipocytes compared to that measured in the mouse macrophage cell line RAW 264.7 (data not shown). Furthermore, exposure to TNF-α resulted in a 2-fold increase in mRNA expression compared to basal levels in 3T3-L1 adipocytes (*P* < 0.05, *n* = 6) (See Supplementary on-line Fig. ii).

### 3.5 Nicotinic acid increases gene expression of the anti-inflammatory adipokine, adiponectin in 3T3-L1 adipocytes

**Fig. 4:**
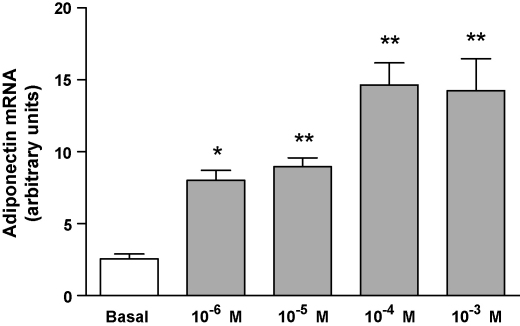
mRNA levels of adiponectin determined by real-time RT-PCR using the 2-ΔΔCT method, normalised to the housekeeping gene cyclophilin. Cells were incubated for 4 h with DMEM only (Basal), DMEM + nicotinic acid 10^−6^–10^−3^ M. *n* = 6 for each treatment, ^**^P < 0.01, ^***^P < 0.001 via one-way ANOVA with Bonferroni's multiple comparison post-hoc test.

We tested whether NA could directly alter expression of adiponectin in adipocytes by analysis of mRNA taken from adipocytes exposed to varying doses of NA, (10^−6^–10^−3^ M) resulted in an increase in adiponectin mRNA in a dose-dependent manner (3–6-fold *n* = 6, *P* < 0.01 after 4 h) (Fig. 4). Total adiponectin protein released into the media was 17–20 ng/mL and was unchanged relative to basal levels regardless of treatment as measured by Luminex™ assay, data not shown.

### 3.6 Nicotinic acid reduces the chemoattractant properties of adipocyte conditioned media

**Fig. 5:**
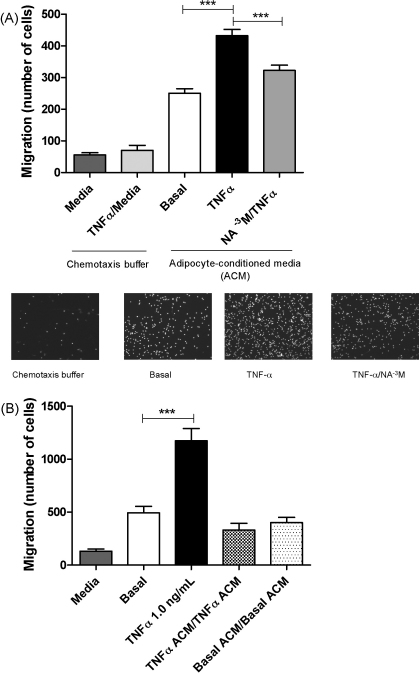
(A) Chemotaxis of mouse macrophages to “adipocyte conditioned media” from 3T3-L1 cells, 4-h treatments with TNF-α and Nicotinic acid. ^***^Basal vs. TNF-α, *P* < 0.001, TNF-α vs. TNF-α and NA^−3^ M. There was no significant difference in cell migration between the chemotaxis buffer alone and chemotaxis buffer with TNF-a 1.0 ng/mL added to the chemotaxis buffer. (B) Chemokinesis of mouse macrophages to “adipocyte conditioned media” (ACM) from 3T3-L1 cells, 4-h treatments with TNF-α and nicotinic acid. ^***^Basal vs. TNF-α, *P* < 0.001. The lower panels show representative images of DAPI fluorescence from the nuclei of migrated macrophages under each of the conditions.

To investigate whether changes in gene expression resulting from nicotinic acid treatment had a physiological effect, we used a chemotaxis assay to measure macrophage migration towards chemo-attractants released by the adipocytes into the surrounding media, ‘adipocyte conditioned media’, (ACM) from differentiated treated 3T3-L1 adipocytes. Neither media alone nor media spiked with TNF-α stimulated chemotaxis (Fig. 5). However, ACM collected from adipocytes that had been exposed to TNF-α provoked an 80% increase in macrophage migration compared to that of basal media collected from unstimulated 3T3-L1 adipocytes. The increase in chemotaxis observed with ACM from TNF-α treated cells was ameliorated by 27% ± 3.5%, (*P* < 0.001) with ACM from TNF-α challenged cells that had been treated with nicotinic acid (Fig. 5). To confirm that the ACM induced true chemotaxis rather than an increase in haptotactic movement due to cellular activation, chemokinesis experiments were carried out. There was no net migration macrophages that were suspended in the ACM collected from TNF-α-stimulated cells and placed over the same concentration of ACM in the transwell (Fig. 5).

## 4 Discussion

This study investigated potential anti-inflammatory actions of NA. The data demonstrate, for the first time that, in adipocytes, NA potently suppresses TNF-α-induced expression and release of the pro-atheorgenic chemokines, MCP-1, RANTES and fractalkine. The reduction in pro-inflammatory chemokine gene expression observed with NA was abolished by pre-treatment with pertussis, indicating that these effects are receptor-mediated via GPCR signalling. Furthermore, the NA treatment was associated with a reduction in macrophage chemotaxis using conditioned media taken from adipocytes treated with TNF-α. These observations demonstrate pleiotropic lipid-independent effects of NA on the release of inflammatory molecules from adipocytes.

NA is well established as a treatment for dyslipidaemia as it has a potent effect on lowering plasma LDL-cholesterol and raising HDL-cholesterol. Numerous clinical studies have demonstrated a significant reduction in cardiac events and cardiovascular disease-related mortality with nicotinic acid treatment [15,18,19]. However, the exact mechanism of action of this drug is still not fully understood. The discovery of a GPCR, GPR109a which binds NA with a high affinity is very highly expressed in adipocytes, has directed research into the anti-lipolytic effects of NA in adipose tissue [12,13]. Acting via GPR109a, NA has been shown to suppress free fatty acid (FFA) release from adipose tissue and it is thought that the subsequent reduction in FFA flux to the liver reduces triglyceride synthesis and production VLDL, by depleting substrate.

In addition to its well-described effects on lipid profiles, several studies have shown that NA treatment increases serum levels of the atheroprotective adipokine adiponectin [16,20]. In a recent study by Linke et al. [21], NA treatment was associated with a significant increase in adiponectin. This study also reported significant reduction in mean adipocyte size, which was coupled to an increase in insulin sensitivity both *in vivo* as judged by euglycaemic-hyperinsulinemic clamp, and by insulin-stimulated glucose transport in isolated subcutaneous adipocytes. Although adiponectin secretion is usually related inversely to adipose tissue mass, the observed increase in adiponectin in NA-treated patients was not associated with any alteration in body mass index (BMI), suggesting a qualitative alteration in the regulation and secretion of adiponectin from adipocytes.

Adipose tissue has the capacity to contribute to both systemic and local (perivascular) inflammation associated with atherosclerosis. Since adipose tissue is an important site of action for NA, we hypothesized that NA could directly affect the inflammatory profile of adipose tissue and that this might be mediated by chemokines and adiponectin.

In the current study, we have demonstrated a dose-dependent increase in gene expression of adiponectin in response to NA treatment after 4 h. This finding supports previous studies showing that serum adiponectin and adiponectin gene expression is increased after treatment with NA in human isolated adipocytes [21]. We have shown elsewhere that serum adiponectin is increased in patients treated for 6 months with extended release nicotinic acid compared to placebo [22] Despite these observations, adiponectin protein secretion was not altered in the current study, after treatment for 24 h with nicotinic acid (10^−4^ and 10^−3^ M). Others have made similar observations in 3T3-L1 adipocytes [20]. This apparent anomaly is possibly due to regulation or deficiency of normal receptor mediated secretory pathways in this cell line. [20]

Under conditions of inflammation associated with cardiovascular disease, as well as an increase in mobilisation of fatty acids from adipose tissue, there is increased secretion of pro-atherogenic, pro-inflammatory adipocytokines and chemokines [23]. In the present study, the chemokines from the CC and CX3 families, MCP-1, RANTES and fractalkine were studied since they contribute significantly to the recruitment of inflammatory T cells and macrophages into atherosclerotic lesions [24]. In addition, by micro-array screening these chemokines have shown not only to be expressed in 3T3-L1 adipocytes but are also upregulated by a 24-h exposure to the T-helper 1 cytokine, interferon-γ [25]. Furthermore, MCP-1 and RANTES play a role in the early progression of atherosclerosis by induction of transendothelial migration via CCR2 and CCR5 chemokine receptors [26].

TNF-α was chosen as the pro-inflammatory stimulus since it is an important cytokine in the progression of atherosclerosis wherein it exerts pro-inflammatory effects on endothelial cells, smooth muscle cells and macrophages [27]. Furthermore, in humans, enhanced TNF-α expression in adipose tissue is associated with insulin resistance and obesity [28]. In addition, TNF-α also plays a crucial role in mediation of the inflammatory process in adipose tissue [29]. Crosstalk between adipocytes and macrophages was elegantly demonstrated by Suganami et al. who reported that co-culture of 3T3-L1 cells and the mouse macrophage cell line, RAW264.7 resulted in upregulation of MCP-1 gene expression in adipocytes, which was abolished by incubation with anti-TNF-α antibody [2].

In the present study we demonstrated that TNF-α treatment at as low a concentration of 0.5 ng/mL resulted in a significant upregulation of MCP-1, RANTES and fractalkine, with the response being maximal at 1 ng/mL. These chemokines are important modulators inflammation that are secreted from adipose tissue and are responsive to the pro-inflammatory cytokine TNF-α thus providing a possible link between inflammation in adipose tissue and the progression of atherosclerosis.

## 5 Conclusions

In this study, we have shown that NA can reduce the inflammatory profile of adipocytes. This may contribute to the overall benefits of NA *in vivo* by reducing potentially harmful effects of within-adipose tissue inflammation and suppressing the contribution of adipose tissue to systemic and perivascular inflammation. These findings demonstrate lipid-independent effects of NA, which could have important implications in the treatment of cardiovascular disease, and warrant further investigation.

## Figures and Tables

**Table 1:** Primer sequences for quantitative real-time RT-PCR.

Gene	Primer sequence
Cyclophilin	Sense, 5′-GGCCGATGACGAGCCC-3′
	Antisense, 5′-TGTCTTTGGAACTTTGTCTGCAA-3′

Adiponectin	Sense, 5′-GTTGCAAGCTCTCCTGTTCC-3′
	Antisense, 5′-ATCCAACCTGCACAAGTTCC-3′

CCL5	Sense, 5′-TCCAATCTTGCAGTCGTGTTTG-3′
	Antisense, 5′-TCTGGGTTGGCACACACTTG-3′

MCP-1	Sense, 5́-TTCCTCCACCACCATGCAG-3́
	Antisense, 5́-CCAGCCGGCAACTGTGA-3́

Fractalkine	Sense, 5′-CCAAGACGCCATGAAGCAT-3′,
	Antisense, 5′-TCAAACTTGCCACCATTTTTAGTG-3′
