# Supplementary material for: Anti-inflammatory effects of nicotinic acid in adipocytes demonstrated by suppression of fractalkine, RANTES, and MCP-1 and upregulation of adiponectin
Source: Atherosclerosis. 2010 Mar;209(1):89–95. doi: 10.1016/j.atherosclerosis.2009.08.045 (PMC2839075; doi:10.1016/j.atherosclerosis.2009.08.045)
Supplement: Supplementary file 1 [file mmc1.pdf]

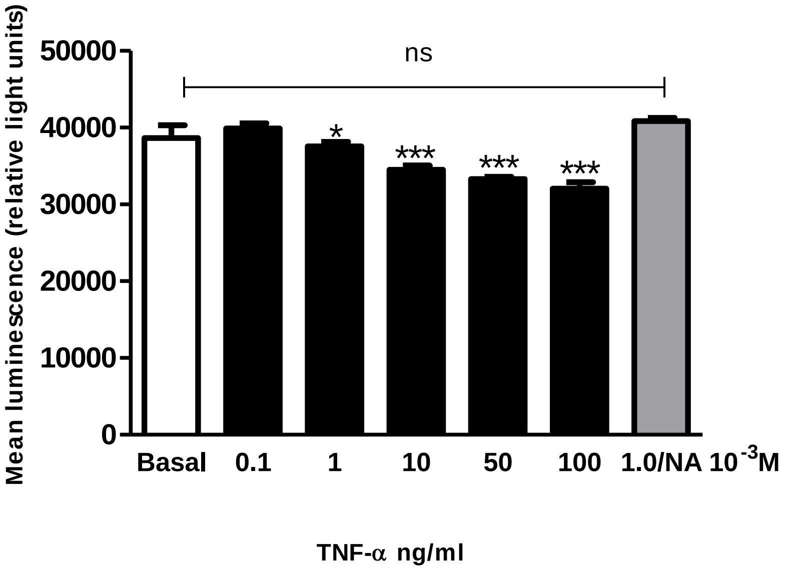

Supplementary
Figure (i)

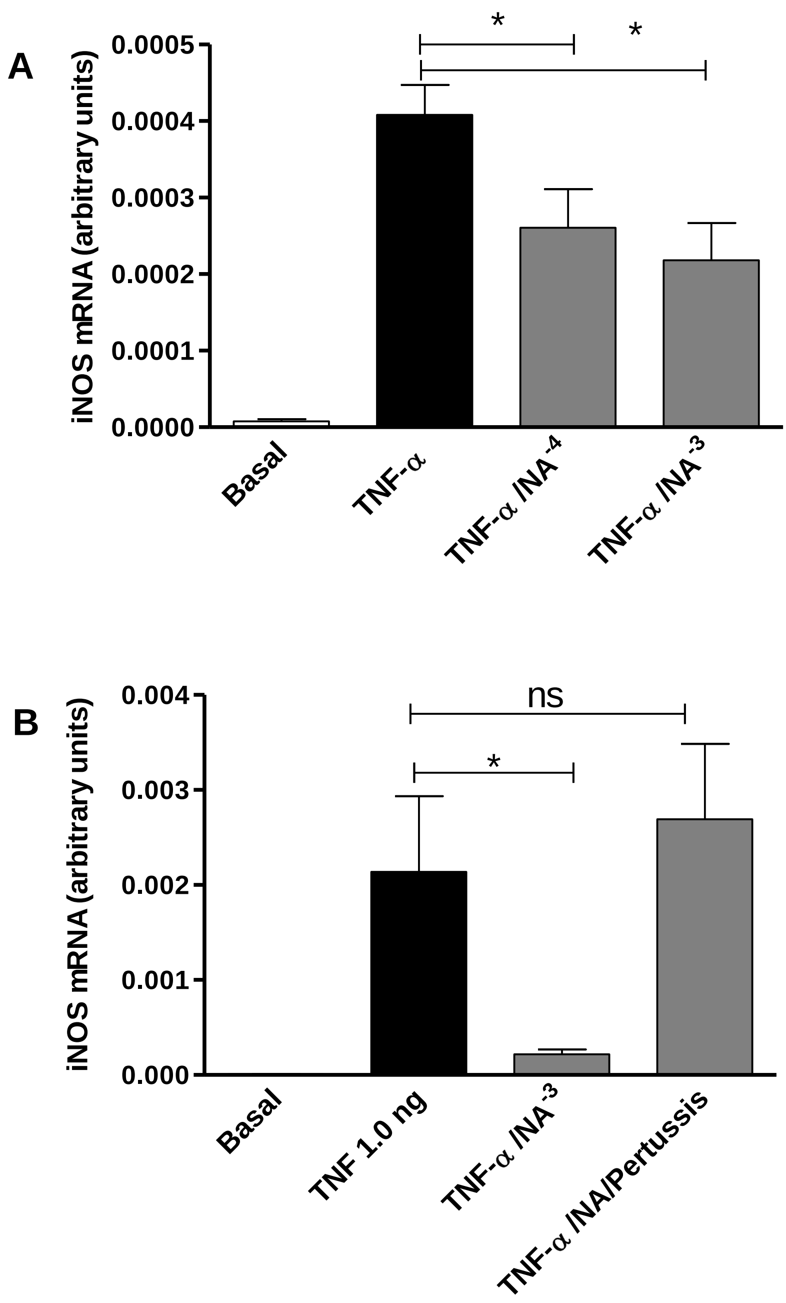

Supplementary
Figure (ii)

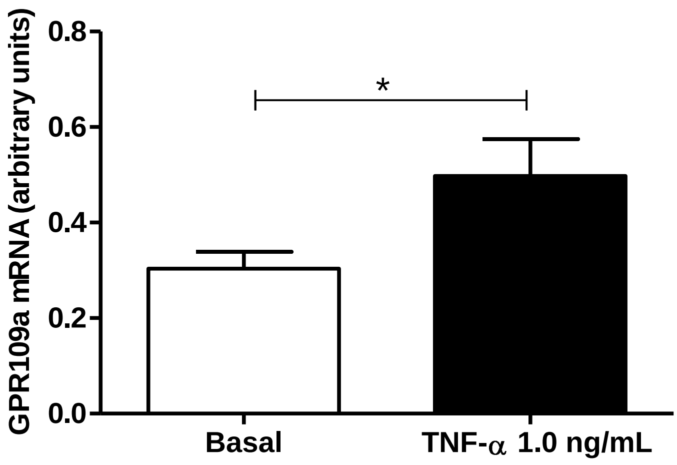

Supplementary
Figure (iii)

Supplementary Methods and Results

Cell viability methods

Cell viability was measured in 3T3-L1 cells differentiated for 10 days then treated with varying doses of TNF-α. Viability was measured using The CellTiter 96® AQ_{ueous} One Solution Cell Proliferation Assay (Promega, Southampton, UK). Absorbance at 490 nm was measured and recorded as mean fluorescence measured by relative light units.

Measurement of iNOS and GPR109a Gene expression in 3T3-L1 Adipocytes

Total RNA was prepared from cell lysates of 3T3-L1 adipocytes differentiated for 10-14 days. Total RNA was purified using Qiagen RNEasy mini columns and 1 μg was reverse transcribed using a QuantiTect® Reverse Transcription Kit containing a genomic DNA wipe-out step and using Oligo dT's and random hexamers as primers. Inducible nitric oxide synthase (iNOS) mRNA expression was measured by Taqman™ Gene Expression assay ID Mm01309902_m1 according to manufacturer's instructions (supplementary figure (ii).

GPR 109a mRNA expression was measured by Real-time PCR using Sybr Green Mastermix (Applied Biosystems, Warrington, UK) and sense and anti-sense primers (0.25 μM final concentration). Primer sequences were sense; 5' ATGAAAACATCGCCAAGGTC 3' and antisense; 5' TGGATTTCCAGGACTTGAGG 3', expected product size, 117 base pairs. Cycling parameters were as follows: activation of Taq polymerase, 10 min at 95°C, then 40 cycles at 95°C for 15 sec, then extension at 60°C for 1 min, followed by a melt curve analysis. PCR products were sequenced to confirm identity using the Illumina

Genome Analysis system. Sequencing results were subjected to BlastN analysis and confirmed 100% identity with mouse GPR109a. To measure a whether TNF−α was able to affect gene expression of GPR109a, differentiated 3T3-L1 adipocytes were incubated with TNF-α 1.0 ng/mL for 4 h and mRNA was measured by quantitative RT-PCR (supplementary figure iii).

Supplementary Figure (i) Cell viability in 3T3-L1 cells treated with varying doses of TNF-α was measured using CellTiter 96® Aqueous One Solution Cell Proliferation Assay (Promega, Southampton, UK). Absorbance at 490 nm was measured and recorded.

Supplementary Figure (ii) A. mRNA levels of iNOS, for RNA analyses, cells were incubated for 4 h and for media analysis cells were incubated for 24 h, with DMEM only (Basal), DMEM + TNF-α 1.0 ng / ml (TNF-α), DMEM + TNF-α 1.0 ng / ml + nicotinic acid 10^{-4} M (TNF-α + NA^{-4}) or DMEM + TNF-α 1.0 ng / ml + nicotinic acid 10^{-3} M (TNF-α + NA^{-3}). N = 6 for each treatment, *$P < 0.05$, via one-way ANOVA with Bonferroni's multiple comparison post-hoc test.

B. The effect of pre-treatment with pertussis (PTX) on adipocyte mRNA expression of iNOS. N=6 for each treatment, *$P < 0.05$.

Supplementary Figure (iii) TNF-α exposure 1.0 ng/mL for 4h upregulates GPR109a mRNA in 3T3-L1 differentiated adipocytes (* $P < 0.05$, n = 6).
